# Navigating the Landscape of Translational Geroscience in Canada: A Comprehensive Evaluation of Current Progress and Future Directions

**DOI:** 10.1093/gerona/glae069

**Published:** 2024-03-15

**Authors:** Guy Hajj-Boutros, Andréa Faust, John Muscedere, Perry Kim, Naji Abumrad, Stéphanie Chevalier, Mylene Aubertin-Leheudre, Howard Bergman, Dawn Bowdish, Jessica Burford, Stacy Carrington-Lawrence, Hélène Côté, David E Dawe, Philipe de Souto Barreto, Colin Farrelly, Robert Fowler, Gilles Gouspillou, Lea Harrington, Sofie Lautrup, Susan Howlett, Mahdi Imani, James Kirkland, George Kuchel, Frédérick A Mallette, José A Morais, John C Newman, Daryl Pullman, Felipe Sierra, Jeremy Van Raamsdonk, Jennifer Watt, Rebecca Jane Rylett, Gustavo Duque

**Affiliations:** Department of Medicine, McGill University and Research Institute of the McGill University Health Centre, Montréal, Québec, Canada; Department of Medicine, McGill University and Research Institute of the McGill University Health Centre, Montréal, Québec, Canada; Schouela Centre of Excellence for Sustainable Health of Seniors (Schouela CEDurable), Montreal, Québec, Canada; Canadian Frailty Network, Kingston, Ontario, Canada; Department of Critical Care Medicine, Queen’s University, Kingston, Ontario, Canada; Canadian Frailty Network, Kingston, Ontario, Canada; Division of Surgical Oncology and Endocrine Surgery, Department of Surgery, Vanderbilt University Medical Center, Nashville, Tennessee, USA; School of Human Nutrition, McGill University, Montréal, Québec, Canada; Département des Sciences de l’Activité Physique, Faculté des Sciences, Université du Québec à Montréal, Montréal, Québec, Canada; Schouela Centre of Excellence for Sustainable Health of Seniors (Schouela CEDurable), Montreal, Québec, Canada; Department of Family Medicine, Faculty of Medicine and Health Sciences, McGill University, Montreal, Québec, Canada; Department of Pathology and Molecular Medicine, McMaster University, Hamilton, Ontario, Canada; Arthur Labatt Family School of Nursing, Faculty of Health Science, Western University, London, Ontario, Canada; Division of Aging Biology, National Institute on Aging, National Institutes of Health, Bethesda, Maryland, USA; Department of Pathology and Laboratory Medicine, University of British Columbia, Vancouver, British Columbia, Canada; Department of Internal Medicine, Max Rady College of Medicine, Rady Faculty of Health Sciences, University of Manitoba, Winnipeg, Manitoba, Canada; Paul Albrechtsen Research Institute, CancerCare Manitoba, Winnipeg, Manitoba, Canada; IHU HealthAge, CERPOP UMR 1295, Toulouse, France; Department of Political Studies, Queen’s University, Kingston, Ontario, Canada; Department of Critical Care Medicine, Sunnybrook Health Sciences Centre, Toronto, Ontario, Canada; Département des Sciences de l’Activité Physique, Faculté des Sciences, Université du Québec à Montréal, Montréal, Québec, Canada; Department of Biochemistry, University of Toronto, Toronto, Ontario, Canada; Department of Clinical Molecular Biology, University of Oslo, and Akershus University Hospital, Lørenskog, Norway; Department of Medicine (Geriatric Medicine), Dalhousie University, Halifax, Nova Scotia, Canada; Australian Institute for Musculoskeletal Science (AIMSS), Geroscience and Osteosarcopenia Research Program, University of Melbourne and Western Health, St Albans, Victoria, Australia; Mayo Clinic, Rochester, Minnesota, USA; UConn Center on Aging, University of Connecticut School of Medicine, UConn Health, Farmington, Connecticut, USA; Department of Medicine, Université de Montréal, Montréal, Québec, Canada; Maisonneuve-Rosemont Hospital Research Centre, Montréal, Québec, Canada; Department of Medicine, McGill University and Research Institute of the McGill University Health Centre, Montréal, Québec, Canada; Buck Institute for Research on Aging, San Francisco, California, USA; Faculty of Medicine, Memorial University, St. John’s, Newfoundland, Canada; Hevolution Foundation, Riyadh, Saudi Arabia; Research Institute of the McGill University Health Centre, Montreal, Quebec, Canada; Department of Critical Care Medicine, Sunnybrook Health Sciences Centre, Toronto, Ontario, Canada; Department of Physiology and Pharmacology, Western University, London, Ontario, Canada; Department of Medicine, McGill University and Research Institute of the McGill University Health Centre, Montréal, Québec, Canada; Schouela Centre of Excellence for Sustainable Health of Seniors (Schouela CEDurable), Montreal, Québec, Canada

**Keywords:** Aging, Animal studies, Clinical trials, Geroscience, Translational Research

## Abstract

The inaugural Canadian Conferences on Translational Geroscience were held as 2 complementary sessions in October and November 2023. The conferences explored the profound interplay between the biology of aging, social determinants of health, the potential societal impact of geroscience, and the maintenance of health in aging individuals. Although topics such as cellular senescence, molecular and genetic determinants of aging, and prevention of chronic disease were addressed, the conferences went on to emphasize practical applications for enhancing older people’s quality of life. This article summarizes the proceeding and underscores the synergy between clinical and fundamental studies. Future directions highlight national and global collaborations and the crucial integration of early-career investigators. This work charts a course for a national framework for continued innovation and advancement in translational geroscience in Canada.

The inaugural Canadian Conference on Translational Geroscience was held as 2 complementary sessions in Montreal on October 6–7, 2023, and in Toronto on October 26, 2023. These sessions brought together interdisciplinary experts and researchers in aging to outline the geroscience landscape in Canada and delve into the relationship between biology and health within the context of aging. By focusing on unraveling the complex connections between fundamental biological processes and well-being, the conferences highlighted key aspects such as cellular senescence, molecular and genetic factors, and their relationship to the development of chronic disease. Importantly, the discourse extended beyond theoretical exploration, emphasizing the pragmatic application of these insights, focusing on how geroscience innovations could enable disease prevention and maintenance of health, function, resilience, and quality of life for older adults. From a person-centered holistic perspective, advances in geroscience will require linkages with initiatives such as the World Health Organization’s Integrated Care for Older People (ICOPE) framework focused on intrinsic capacity and prevention of frailty (1,2) if they are to improve the lives of older adults. The conferences also highlighted the early stage and developing nature of geroscience and the need to establish and propel the field of geroscience forward in Canada. A key conference finding was that most Canadian investigators in aging do not necessarily identify as working in the field of geroscience. The consensus at the conferences was that there is a critical need to establish additional avenues for geroscience research and venues for interdisciplinary collaborations, both nationally and internationally.

The conferences were developed in recognition that geroscience is rapidly progressing and, unless there are coordinated efforts to foster its development, scientific communities both national and local risk being left behind. It came together with funding from federal, pan-Canadian, and provincial organizations such as the Canadian Institutes of Health Research Institute of Aging (CIHR-IA), the Canadian Frailty Network (CFN, www.cfn-nce.ca), and the *Réseau Québécois de Recherche sur le Vieillissement* (RQRV). These organizations, along with other funders for the study of aging, will play a pivotal role in catalyzing, propelling, and sustaining impactful geroscience research initiatives by convening researchers, clinicians, patients, caregivers, and other stakeholders to shape the evolving landscape of research in aging and implementation into practice. Significant resources for the conduct of research on aging exist in Canada, such as the longitudinal studies Canadian Longitudinal Study on Aging (CLSA) and the Nutrition as a Determinant of Successful Aging (NuAge) that will provide rich data sets for investigations into aging-related phenomena. Additionally, this manuscript outlines future directions discussed at the conferences, highlighting the need for collaborative efforts among researchers, organizations, and funders in Canada and across the globe to advance the field of geroscience. It underscores the need to capacitate early-career geroscience investigators, thus ensuring a seamless transition of knowledge and expertise to the next generation.

## Bridging Clinical and Fundamental Research in Aging

Research into the biology of aging has matured to the point where it is ready to move out of the laboratory into clinical research. Further, there is a need to rigorously test the geroscience hypothesis, which posits that the major risk factor for chronic disease is aging and that modifying aging biology will improve the health span (3). These were the underlying themes of the conferences. Conference participants and researchers engaged in discussions, delving into the facets of aging and the unique opportunities and challenges presented by the relatively newly emerging field of geroscience. Presentations and subsequent discussion highlighted critical insights into the aging process and associated challenges, including the use of animal models (eg, Caenorhabditis *elegans,* mice) to test novel hypotheses in the geroscience field, including their translation into human aging.

Nutrition emerged as a focal point, with discussions spanning from the impact of dietary patterns on aging to the role of specific nutrients and supplements, such as protein intake, leucine, and β-hydroxy-β-methyl-butyrate (HMB) in promoting health and longevity (4,5). Outlined were the intricate connections between nutritional choices and their repercussions on cellular aging, chronic diseases, and overall well-being in older people (6). In addition, the role of activity and physical exercise in promoting healthy aging was emphasized, given its profound impact on maintaining muscle mass, cognitive function, and cardiovascular health (7). A key understanding was that any future study of geroscience interventions would need to account for the underlying nutritional and exercise characteristics of the population being studied.

Cognitive aging and its myriad dimensions were also a major part of the discussion. Presentations delved into the mechanisms underlying age-related cognitive decline, exploring interventions to improve care, programs, and services to preserve cognitive function. They further outlined the complex cross-talk between the brain and other organs (ie, gut, muscle, and bone) and the intricate relationship between brain health and aging (8).

An important discussion point during the conferences was the intersection between aging and cancer. The complex dynamics between aging processes and the development of cancer were illuminated (9). Further deliberations explored strategies for addressing cancer risk in older populations, the need to better understand the unique challenges of cancer treatment in older adults, and the potential of geroscience interventions for cancer prevention (10).

The role of medications as geroscience interventions was also highlighted, focusing on understanding the impact of interventions such as metformin (11) on age-related diseases. Participants discussed novel approaches to drug development, considering the unique physiological changes accompanying aging and tailoring treatments to optimize efficacy and minimize side effects in older adults. Given the difficulties inherent in the drug development process, including the uncertain regulatory process for geroscience medications, repurposing and studying currently approved pharmaceuticals or nutraceuticals was felt to be the easiest path forward. In addition, there was discussion on the advancement of senolytics, a class of molecules that selectively kill senescent cells, thus slowing the aging process and development of morbidity in preclinical models (12,13). The U.S.-based Translational Geroscience Network (gerosciencenetwork.org) is currently associated with over 80 geroscience intervention clinical trials, 30 of which involve senolytics. Further discussions explored the complex landscape of chronic diseases in aging, spanning from diabetes (14), cardiovascular diseases (15), and neurodegenerative conditions (16). Researchers shared insights into the common underlying mechanisms linking chronic diseases and aging, potentially paving the way for holistic approaches to disease prevention and management in older populations.

The conferences featured contributions from early-career investigators and recipients of CIHR-IA’s first geroscience grant competition, injecting fresh perspectives into the dialogue. These presentations featured innovative studies across various domains, showcasing cutting-edge studies advancing our understanding of aging and contributing to the evolving landscape of geroscience. Topics included the effect of bedrest on muscle mass and bone vascularization (17,18); nutritional strategies to prevent increasing inflammation (19) and changes in muscle morphology on a cellular level with aging (20); association of chronic inflammation with late-life health and frailty (21,22); the potential impact of chronic and latent viral infections on biological aging (23,24); contribution of mitochondrial dysfunction to aging-related loss of muscle mass and function (25–28) span; CRISPR-mediated chemical and genetic interrogation of senotherapeutics (29,30); novel insights into mechanism-of-action for a new class of senolytics to treat age-related pathologies (31–35); understanding the aging process to develop novel treatments for neurodegenerative disease (36–40); and linking inflammation and metabolism with frailty in chronic cancer (41–44).

Overall, the conferences weaved together the threads of nutrition, exercise, cognition, cancer, medication, chronic diseases, and the invaluable contributions of early-career investigators. The collaborative exchange of knowledge and ideas not only illuminated the current state of geroscience but also paved the way for future avenues of research that will shape the future trajectory of geroscience and aging research in Canada.

## Funding for Geroscience Research

Building upon the foundational conference discussions is the need to coordinate with health research funding agencies. In Canada, the CIHR, as the foremost supporter of health research, has been instrumental in fostering investigations at the crossroads of basic and clinical sciences. CIHR consists of 13 member institutes that work together to deliver its mandate for scientific excellence and the creation of new knowledge to improve health for Canadians. One of its member institutes is the CIHR-IA, whose goal is to improve the quality of life and health of older Canadians by understanding and addressing or preventing the consequences associated with aging; they will be a key partner advocating for funding of geroscience research moving forward. The CFN, a pan-Canadian not-for-profit organization, also addresses aging and frailty-related challenges and facilitates translational efforts to enhance the health and well-being of older adults. Its focus is on the prevention of aging-related morbidity and frailty, and geroscience offers much promise in this regard. As a national organization, they will be important drivers for the coordination of geroscience in Canada. In Quebec, the Fonds de Recherche du Québec – Santé (FRQS) has been a linchpin in advancing aging research, providing crucial support for projects that span the spectrum from foundational studies to clinical applications. Additionally, with its collaborative framework, the RQRV has amplified the impact of aging research by fostering interdisciplinary initiatives and knowledge exchange. The collective contribution of these funding agencies amounts to a substantial investment in advancing our understanding of aging.

This investment has not only propelled research projects forward but has catalyzed collaborative endeavors, enabling the translation of basic science discoveries into tangible clinical interventions that improve care of Canada’s aging population.

Collaboration between Canadian funding agencies supporting aging research and the National Institute on Aging (NIA) in the United States has further enriched the landscape of translational geroscience. The CIHR-IA, CFN, and FRQS have actively engaged in cross-border initiatives, fostering a robust exchange of knowledge and resources with the NIA (1,45). This collaborative synergy extends beyond geographical boundaries, exemplifying a shared commitment to advancing our understanding of aging. The strategic alignment of research priorities between these Canadian agencies and the NIA has resulted in joint initiatives, cooperative studies, meetings, and reciprocal support for groundbreaking projects (15). Such collaboration with international collaboration not only amplifies the impact of individual endeavors but also accelerates the pace of discoveries in the field of aging research (1,45). The shared objective is to strategically broaden the scope of data collection across countries, capitalizing on diverse populations and the ability to achieve larger sample sizes. This approach not only enhances the scientific rigor of the studies but also contributes to a more comprehensive understanding of the research questions at hand. It is only with multinational collaboration, as well as the support and vision of both national and international agencies, that scientific inquiry into unraveling the complexities of aging and development of novel interventions targeting enhancement of the quality of life for older adults will go forward.

## Longitudinal Databases in Canada: A Wealth of Insight Into Aging

In Canada, several longitudinal databases on aging exist that will be key to understanding the aging process, the study of the geroscience hypotheses, and supporting the design of geroscience clinical interventions.

### Canadian Longitudinal Study on Aging

Initiated in 2010, the CLSA (www.clsa-elcv.ca) is a cornerstone in Canadian aging research. Encompassing over 50 000 participants aged 45–85, this comprehensive study explores a myriad of factors, including health, lifestyle, and socioeconomic determinants. The longitudinal nature of the CLSA allows for the examination of how these variables evolve over time, offering invaluable insights into the aging process and its multifaceted aspects. As of 2022, the CLSA has been the focus of more than 500 research projects completed or currently underway in Canada and worldwide, all of which remain readily accessible for future research endeavors. Sustained funding for the CLSA has allowed and will continue to allow follow-up with the same cohort of individuals over many years ensuring a rich and evolving data set that contributes to a deeper understanding of aging (46–48).

### Quebec Longitudinal Study on Nutrition and Successful Aging

The NuAge (http://www.rqrv.com/en/init_NuAge.php) study, launched in 2003, studies the nutritional dimensions of aging, particularly within the province of Quebec. By collecting data on dietary intake and patterns, health outcomes, and lifestyle choices, NuAge aims to unravel the links between nutrition and aging contributing to our understanding of how dietary factors influence the health trajectories of older adults. To the present, the database has generated more than 88 publications. Ongoing funding for this database enables a 15-year follow-up with the cohort survivors spanning multiple years focusing on nutrition, body composition, and cognition (49,50).

### CARTaGENE

CARTaGENE (cartagene.qc.ca) stands as a prominent public research initiative hosted by the CHU Sainte-Justine, with the primary objective of catalyzing advancements in health research. The foundation of CARTaGENE revolves around a comprehensive collection that integrates biological samples with intricate data sets related to the health profiles and lifestyle patterns of a significant cohort. At its inception, CARTaGENE embarked on a recruitment drive that successfully enlisted 43 000 individuals from the province of Quebec. These participants, falling within a specific age bracket of 40 to 69 years, provide a diverse and expansive pool of information, facilitating various avenues of health-related investigations at early stages. The biobank aspect of CARTaGENE encompasses a vast repository of biological specimens, which include but are not limited to, DNA samples, blood components, tissues, and other pertinent biological materials. These samples are meticulously stored under optimal conditions, ensuring their integrity and viability for extensive research endeavors. Through its expansive collection and collaborative ethos, CARTaGENE continues to drive advancements, fostering a deeper understanding of health dynamics and paving avenues for improved healthcare strategies and interventions.

### National Population Health Study of Neurological Conditions

While not exclusively dedicated to aging, the National Population Health Study of Neurological Conditions (NPHSNC), which commenced in 2009, captures essential data on neurological conditions in the Canadian population. Given the increased prevalence of neurological conditions with age, the NPHSNC offers a valuable lens into the neurological aspects of aging, enriching the overall landscape of longitudinal studies in Canada.

### Health and Retirement Study—Canadian Sample

Extending the renowned Health and Retirement Study (HRS) from the United States, the Canadian sample of HRS began in 1992. Focused on individuals aged 51 and older, this study delves into the dynamics of aging, retirement, and health. By harmonizing with the U.S. HRS, the Canadian Sample facilitates cross-national comparisons, broadening the scope of insights into aging-related phenomena (51–53).

These longitudinal studies and associated databases collectively form a robust infrastructure for aging and geroscience research in Canada, providing researchers with a wealth of data to investigate the complex interplay of genetic, biological, social, and environmental factors shaping the aging process.

## Empowering the Future: Integrating Early-Career Investigators Into Aging Research

As we stand on the precipice of a demographic shift toward an aging population (54), it is imperative to cultivate a cohort of early-career investigators who are not only passionate about understanding the complexities of aging but are also equipped with the skills and resources to pioneer transformative translational research in geroscience. Capacitating early-career investigators into geroscience research is a necessity to advance geroscience in Canada and to address the evolving healthcare needs of an increasingly aged global population. Discussion at the conferences included organizational structures and frameworks that currently exist for other areas of science/medicine (eg, Canadian Training Platform for Trials Leveraging Existing Networks [CANTAP] Talent, https://www.cantaptalent.ca/) that could be used as a template for geroscience organizations to facilitate training, educating, and mentoring the next generation of interdisciplinary geroscience researchers. These types of organizational structures and supports could help with the following:

1. Support for Early-Career Investigators:

A pivotal strategy to foster the integration of early-career investigators into aging and geroscience research is to ensure adequate funding for graduate, postdoctoral, and early-career researchers. Financial support specifically directed toward early-career researchers is critical to launching independent research programs and establishing a critical mass of aging and geroscience researchers in Canada. Increased funding for early-career investigators will not only facilitate groundbreaking research but also send a clear message about the significance society places on addressing aging-related challenges.

2. Courses on Geroscience During Undergraduate and Medical School Education:

Attendees agreed that introducing courses on aging and translational geroscience research to undergraduate and medical school students is a key component of building Canada’s basic and clinical research capacity. Incorporating aging-related coursework and research opportunities at these crucial junctures not only exposes budding scientists and clinicians to the intricacies of aging and geroscience but also instills a deeper understanding of how age-driven health and well-being can be achieved. Early exposure will create a pipeline of researchers and clinicians who are passionate about and committed to the field throughout their careers.

3. The Role of Health Professionals:

Emphasizing the preventive aspect of aging is crucial, and health professionals (eg, physicians, nurses, and physiotherapists) play a pivotal role in this process. Integrating early-career health professionals into aging research equips them with the knowledge and tools to focus not only on treating age-related diseases but also on preventing them. Preventive strategies, guided by rigorous clinical research, have the potential to reshape healthcare by enhancing the quality of life for older adults and alleviating the burden on healthcare systems. With this growing body of knowledge and the increasing need to translate bench discoveries to the bedside, there is a need to train the next generation of clinician scientists focused on aging and clinical research. Clinician scientists are clinicians who undertake research training in addition to their clinical roles and dedicate a substantial part of their careers to research (www.royalcollege.ca). The dual role allows for contributions to new knowledge from research questions relevant to clinical practice and its implementation into practice. Networks focused on translational research could provide mentorship and training opportunities in order to develop clinician scientists who are capable of designing and leading effective geroscience clinical trials and submitting competitive research grants for funding. These early-career clinician researchers could be from a wide variety of medical and health professional disciplines, from oncology and critical care to nursing and physical therapy, reflecting the broad potential applicability of geroscience across organ systems and disciplines.

The expanding demographic of older adults demands innovative solutions, from developing personalized treatment, including pharmacologic therapies, to exploring novel technologies (55). Integrating early-career investigators into the field of geroscience research is not just an investment in the future; it is an essential step in ensuring the health and well-being of an aging global population.

## Fostering Collaboration: A Pillar for Advancing Geroscience Research

In the dynamic landscape of geroscience research, the importance of collaboration cannot be overstated. The challenges posed by the aging global population demand a collective and synergistic effort from interdisciplinary researchers worldwide (56). Collaborative work not only enhances the quality of research but also accelerates the translation of discoveries into interventions that improve the lives of older adults.

Interdisciplinary collaboration brings together diverse experiences, perspectives, expertise, and methodologies. This diversity enriches the research landscape, fostering a holistic approach to addressing the multifaceted challenges of aging. Whether it’s sharing insights on cultural nuances, genetic variations, or healthcare practices, interdisciplinary collaborative efforts ensure a comprehensive understanding that transcends preconceived biases and geographical boundaries. In addition, global collaboration allows researchers to pool resources, enabling large-scale, comprehensive studies that wouldn’t be feasible on an individual or regional level. Shared databases, collaborative clinical trials, and joint initiatives leverage the strengths of multiple research teams, facilitating more extensive and robust investigations into the various dimensions of geroscience.

Translating geroscience research findings into real-world applications can be expedited through interdisciplinary collaboration. When researchers from different disciplines work together, translating discoveries into interventions becomes more efficient by anticipating barriers that may not be apparent to one discipline. This acceleration is crucial in ensuring that innovative solutions reach older adults rapidly, addressing their evolving needs and improving health outcomes. In addition to the advantages stated above, there is an influence of researchers’ collaboration on global health policy (57). Combined evidence from diverse research initiatives provides a more comprehensive foundation for shaping policies that affect aging populations worldwide. From preventative strategies (58) to healthcare delivery models (59), collaborative research can contribute to the development of effective, evidence-based policies. Collaboration, whether between disciplines, across the country or internationally not only benefits established researchers but also plays a pivotal role in training the next generation of scientists. Exposure to diverse research environments, mentorship from nonlocal experts, and participation in collaborative projects better equip emerging researchers to deal with the challenges and complexities posed by research aging (60).

The challenges posed by aging populations are not confined to specific regions. Issues such as age-related diseases (61) and disparities in access to healthcare and rate of biological aging due to socioeconomic factors (62) transcend borders. To this end, conference delegates were introduced to, and asked to consider, the social and ethical implications of developing geroscience interventions that aim to reduce multimorbidity, increase health span, and potentially lifespan, on a warming planet of over 8 billion people (60,63). Discussion centered on how aging and climate change are intricately connected and how the goals of climate change and geroscience are complimentary (eg, promoting health and redressing health disparities). Further, it was emphasized that geroscience interventions that increase the resilience of older individuals will be important to facilitate population climate change adaptation.

In summary, collaborative work stands as a cornerstone in advancing geroscience research. The synergies created through interdisciplinary, national, and global collaboration not only deepen our understanding of aging but also enhance the impact of research on the lives of older adults. Collaboration is a powerful catalyst for developing innovative solutions for aging and a future where older adults age with dignity and well-being.

## The Canadian Network on Translational Geroscience: Next Step

Following the success of the Translational Geroscience Network in the United States (gerosciencenetwork.org) and the efforts of the NIA (45) and groups like the International Geroscience Translational Clinical Trials Task Force (1,11), one of the conclusions of the conferences was the need for a Canadian research network in the field of geroscience, which will facilitate collaborations, sharing of resources, and communication among researchers interested in geroscience from bench to bedside and to the community. This Canadian initiative would align with other similar efforts underway in Europe, the United Kingdom, and Japan that are on the path to creating their own networks (64). Delegates, which included members from CIHR-IA, RQRV, and CFN, discussed how a major outcome of the conferences could be the creation of an investigator-led Canadian geroscience clinical trials group focused on investigator-initiated clinical and basic science research, structured in an interdisciplinary collaborative manner. It would provide a national forum for continuing education on geroscience, research in aging, research methodology, and the mentoring of the next generation of clinician scientists. The geroscience clinical trials group would be similar to other clinical trial groups around the world. During the conferences, a Canadian exemplar, the Canadian Critical Care Trials Group (CCCTG) was discussed as an organization to be emulated. The CCCTG was created in 1989 and has over 400 interprofessional members (ie, patient partners, clinical and basic scientists, healthcare professionals, research coordinators, and trainees). It uses a group approach to improve research programs adopted by the CCCTG, thereby leveraging its collective expertise to improve the underlying science, increase its competitiveness at peer review funding, and increase the impact of its work. Further, it conducts training and mentoring of trainees and early-career researchers. With the numerous practice-changing studies conducted under its auspices, it has become highly emulated around the world.

To envision what an ideal Translational Geroscience Network might look like, conferences delegates engaged in focused small group discussions, then convened as one large group. The discussions identified vital components of an ideal network. The small groups followed a strategic evaluative approach that identified key considerations in the following domains: strengths, weaknesses, benefits, and dangers of creating and maintaining a network. Key considerations from each small group revealed many similarities in focus but also uncovered unique aspects. As the large group discussion evolved, a number of final consensus points were identified for establishing an ideal Canadian geroscience network. These are listed below (please note the order of appearance does not denote a ranking).

- Establish a clear and inclusive definition of geroscience that challenges the current healthcare paradigm by focusing less on sickness and disease, and more on health and well-being (ie, expansion of health span) as we age.
- Cocreation and coleadership of an interdisciplinary inclusive research community network that connects patients, clinicians, scientists, and other stakeholders from all relevant disciplines to synergize, and build off each other’s strengths for the conduct research that aligns with the unique needs of all older Canadians.
- Incorporate the diversity of Canada’s multicultural and multilingual populations to address diversity, equity, and inclusion of patients/participants within the Network to reflect the broad Canadian aging population.
- Create an ethical framework and a mindful approach to the importance of the social determinants of health, avoidance of ageism, interplay with climate change, and other dynamics that influence the aging process in the Canadian context.
- Create and retain talent in Canada by establishing processes for mentorship, education, training, and access to resources for the next generation of geroscientists.
- Create centralized supports for: (i) research ethics approvals to expedite research and improve efficiency; (ii) communications to both improve health literacy of all Canadians and help inform/educate Canadians on the importance and potential impact of geroscience research; and (iii) establish other resources like shared biobanks and data science supports.
- Create and implement knowledge translation strategies to move research evidence into healthcare practice and influence healthcare policy.
- Create meaningful relationships with geroscience networks and like-minded organizations from across Canada and globally to establish partnerships and collaborations to share research methodologies, findings, and organizational best practices that ultimately advance geroscience research and increase chances of sustainability.
- Advocate with regulatory agencies for the development and pathways for licensing of geroscience interventions, including pharmaceuticals.

## Conclusion

The inaugural Canadian Conferences on Translational Geroscience have created an indelible mark on the landscape of Canadian geroscience research, spanning from lab-based biological research to clinical research exploring the enhancement of well-being in older adults. This manuscript reports on the collaborative spirit between basic mechanistic and clinical researchers, acknowledging the indispensable contributions of funding bodies—CIHR-IA, CFN, RQRV, and FRQS. Their sustained support has not only propelled impactful aging and geroscience research initiatives forward but has also fostered a collaborative environment, amplifying the impact of aging studies and geroscience research. Longitudinal studies, exemplified by the CLSA, and the CARTaGENE and NuAge studies, will be key resources to gain further insights into the aging process. Looking to the future, the integration of early-career investigators into geroscience research is paramount. Increased funding, early exposure during undergraduate and graduate education, and a focus on prevention by health professionals are strategic initiatives that will ensure an influx of fresh perspectives and innovative solutions to meet the challenges posed by an aging population. Furthermore, collaborative efforts among Canadian funding agencies and the NIA in the United States underscore the global nature of aging research. Joint initiatives and international collaborations have yielded a substantial body of research, reflecting a shared commitment to advancing our understanding of geroscience. The evolving dynamic landscape of geroscience will be marked by collaboration, innovation, and a collective dedication to addressing the multifaceted challenges of aging. As we plan for a future geroscience translational network, armed with insights from these conferences and fueled by ongoing support, we embark on a journey toward a future where aging is characterized by dignity, well-being, and a wealth of opportunities for all.
